# Supplementary material for: Body composition patterns among normal glycemic, pre-diabetic, diabetic health Chinese adults in community: NAHSIT 2013–2016
Source: PLoS One. 2020 Nov 4;15(11):e0241121. doi: 10.1371/journal.pone.0241121 (PMC7641370; doi:10.1371/journal.pone.0241121)
Supplement: S1 Table — (n = 1358)–sensitivity analysis. (DOCX) [file pone.0241121.s001.docx]

| **S1 Table. The body composition markers that related obesity according to diabetes mellitus diagnosis. (n=1358) –sensitivity analysis** | | | | | | | | |
| --- | --- | --- | --- | --- | --- | --- | --- | --- |
| Markers, unit | DM (Self-report or Glucose>=126 or HbA1c>=6.5) | | | | P value | Reg. coefficient (DM vs non-DM)* | | P value |
|  | Yes (n=214) | | No (n=1144) | |  | Beta | 95% CI |  |
|  | mean | SD | mean | SD |  |  |  |  |
| Weight, cm | 69.23 | 13.61 | 63.83 | 12.64 | <0.001 | 3.178 | (1.556 - 4.801) | <0.001 |
| BMI, kg/m^2^ | 26.44 | 4.12 | 24.11 | 3.79 | <0.001 | 1.199 | (0.643 - 1.755) | <0.001 |
| Waist, cm | 91.82 | 10.51 | 83.00 | 10.69 | <0.001 | 3.866 | (2.424 - 5.308) | <0.001 |
| Total fat mass, g | 22447.3 | 7670.89 | 19392.32 | 7049.15 | <0.001 | 1706.092 | (592.837 - 2819.347) | 0.003 |
| Total lean mass, g | 43318.27 | 8426.28 | 41022.61 | 8836.64 | <0.001 | 1542.602 | (776.143 - 2309.062) | <0.001 |
| Total region fat, % | 32.46 | 7.60 | 30.62 | 8.41 | 0.006 | 0.799 | (-0.325 - 1.923) | 0.164 |
| Total tissue fat, % | 33.67 | 7.77 | 31.82 | 8.65 | 0.008 | 0.828 | (-0.329 - 1.985) | 0.161 |
| Fat body Weight, % | 32.03 | 7.60 | 30.18 | 8.39 | 0.007 | 0.008 | (-0.004 - 0.019) | 0.181 |
| Limb fat body Weight, % | 11.82 | 3.80 | 12.60 | 4.17 | 0.005 | -0.002 | (-0.006 - 0.003) | 0.467 |
| Trunk fat body Weight, % | 19.05 | 4.39 | 16.44 | 4.87 | <0.001 | 0.010 | (0.002 - 0.017) | 0.009 |
| Lean body Weight, % | 62.98 | 7.20 | 64.51 | 8.15 | 0.019 | -0.004 | (-0.014 - 0.005) | 0.373 |
| Limb lean body Weight, % | 27.39 | 3.40 | 28.48 | 4.13 | 0.001 | -0.004 | (-0.008 - 0.000) | 0.080 |
| Trunk lean body Weight, % | 30.69 | 3.96 | 30.81 | 4.10 | 0.623 | 0.001 | (-0.004 - 0.007) | 0.691 |
| Limb in fat, % | 36.49 | 5.26 | 41.58 | 6.24 | <0.001 | -0.017 | (-0.023 - -0.010) | <0.001 |
| Trunk in fat, % | 59.76 | 5.30 | 54.39 | 6.60 | <0.001 | 0.019 | (0.011 - 0.027) | <0.001 |
| Limb in lean, % | 43.52 | 2.67 | 44.11 | 2.55 | 0.01 | -0.003 | (-0.006 - 0.001) | 0.128 |
| Trunk in lean, % | 48.71 | 2.35 | 47.77 | 2.06 | <0.001 | 0.005 | (0.002 - 0.008) | 0.001 |

Tested by Mann-Whitney U test or Kruskal-Wallis H test and expressed as mean (SD). DM, diabetes mellitus.

*Tested by generalized linear model and adjusted age, sex, systolic blood pressure, diastolic blood pressure, triglycerides, and HDL. (n=1277)
