# Supplementary material for: Body composition patterns among normal glycemic, pre-diabetic, diabetic health Chinese adults in community: NAHSIT 2013–2016
Source: PLoS One. 2020 Nov 4;15(11):e0241121. doi: 10.1371/journal.pone.0241121 (PMC7641370; doi:10.1371/journal.pone.0241121)
Supplement: S2 Table — (n = 1358)–sensitivity analysis. (DOCX) [file pone.0241121.s002.docx]

| **S2 Table. The body composition markers that related obesity according to diabetes mellitus diagnosis. (n=1358) –sensitivity analysis** | | | | | | | | | | |
| --- | --- | --- | --- | --- | --- | --- | --- | --- | --- | --- |
| Markers, unit | DM (Self-report or Glucose>=126 or HbA1c>=6.5) | | | | | | P value | P value | P value | P value |
|  | Yes with treatment (n=109) | | Yes without treatment (n=105) | | Negative (n=1144) | |  | T vs UT | T vs N | UT vs N |
|  | mean | SD | mean | SD | mean | SD |  |  |  |  |
| Weight, cm | 67.52 | 12.60 | 71.01 | 14.42 | 63.83 | 12.64 | <0.001 | 0.093 | 0.002 | <0.001 |
| BMI, kg/m^2^ | 26.03 | 3.91 | 26.87 | 4.31 | 24.11 | 3.79 | <0.001 | 0.143 | <0.001 | <0.001 |
| Waist, cm | 91.34 | 10.39 | 92.31 | 10.66 | 83.00 | 10.69 | <0.001 | 0.413 | <0.001 | <0.001 |
| Total fat mass, g | 21709.46 | 7488.79 | 23213.30 | 7817.42 | 19392.32 | 7049.15 | <0.001 | 0.147 | 0.002 | <0.001 |
| Total lean mass, g | 42335.51 | 7687.81 | 44338.47 | 9054.25 | 41022.61 | 8836.64 | <0.001 | 0.129 | 0.057 | <0.001 |
| Total region fat, % | 32.20 | 7.72 | 32.74 | 7.49 | 30.62 | 8.41 | 0.021 | 0.663 | 0.081 | 0.022 |
| Total tissue fat, % | 33.39 | 7.88 | 33.96 | 7.69 | 31.82 | 8.65 | 0.027 | 0.647 | 0.096 | 0.025 |
| Fat body Weight, % | 31.72 | 7.70 | 32.35 | 7.52 | 30.18 | 8.39 | 0.021 | 0.553 | 0.096 | 0.019 |
| Limb fat body Weight, % | 11.60 | 3.69 | 12.04 | 3.93 | 12.60 | 4.17 | 0.015 | 0.523 | 0.012 | 0.102 |
| Trunk fat body Weight, % | 18.92 | 4.57 | 19.19 | 4.21 | 16.44 | 4.87 | <0.001 | 0.695 | <0.001 | <0.001 |
| Lean body Weight, % | 63.17 | 7.39 | 62.78 | 7.02 | 64.51 | 8.15 | 0.061 | 0.828 | 0.129 | 0.052 |
| Limb lean body Weight, % | 27.10 | 3.36 | 27.70 | 3.43 | 28.48 | 4.13 | 0.002 | 0.210 | 0.001 | 0.106 |
| Trunk lean body Weight, % | 31.08 | 4.06 | 30.29 | 3.83 | 30.81 | 4.10 | 0.315 | 0.178 | 0.542 | 0.176 |
| Limb in fat, % | 36.21 | 5.01 | 36.78 | 5.51 | 41.58 | 6.24 | <0.001 | 0.478 | <0.001 | <0.001 |
| Trunk in fat, % | 59.89 | 5.07 | 59.62 | 5.56 | 54.39 | 6.60 | <0.001 | 0.763 | <0.001 | <0.001 |
| Limb in lean, % | 42.93 | 2.51 | 44.13 | 2.70 | 44.11 | 2.55 | <0.001 | 0.001 | <0.001 | 0.657 |
| Trunk in lean, % | 49.17 | 2.21 | 48.23 | 2.40 | 47.77 | 2.06 | <0.001 | 0.003 | <0.001 | 0.091 |

Tested by Mann-Whitney U test or Kruskal-Wallis H test and expressed as mean (SD). DM, diabetes mellitus.

*Tested by generalized linear model and adjusted age, sex, systolic blood pressure, diastolic blood pressure, triglycerides, and HDL. (n=1277)
