# Supplementary material for: Body composition patterns among normal glycemic, pre-diabetic, diabetic health Chinese adults in community: NAHSIT 2013–2016
Source: PLoS One. 2020 Nov 4;15(11):e0241121. doi: 10.1371/journal.pone.0241121 (PMC7641370; doi:10.1371/journal.pone.0241121)
Supplement: S3 Table — (n = 1358)–sensitivity analysis. (DOCX) [file pone.0241121.s003.docx]

| **S3 Table. The body composition markers that related obesity according to DM diagnosis. (n=1358) –sensitivity analysis** | | | | | | | |  |
| --- | --- | --- | --- | --- | --- | --- | --- | --- |
| Markers, unit | Reg. Coefficient  (DM with treatment vs Normal)* | | P value | Reg. coefficient  (DM without treatment vs Normal)* | | P value | |  |
|  | Beta | (95% CI) |  | Beta | (95% CI) |  | |  |
| Weight, cm | 2.982 | (0.872 - 5.091) | 0.006 | 3.391 | (1.202 - 5.580) | | 0.002 | |
| BMI, kg/m^2^ | 1.110 | (0.383 - 1.838) | 0.003 | 1.293 | (0.544 - 2.041) | | 0.001 | |
| Waist, cm | 3.791 | (1.896 - 5.687) | 0.000 | 3.943 | (2.015 - 5.872) | | 0.000 | |
| Total fat mass, g | 1560.42 | (116.61 - 3004.23) | 0.034 | 1867.92 | (342.64 - 3393.19) | | 0.016 | |
| Total lean mass, g | 1618.83 | (623.02 - 2614.64) | 0.001 | 1459.95 | (431.71 - 2488.19) | | 0.005 | |
| Total region fat, % | 0.661 | (-0.811 - 2.132) | 0.379 | 0.945 | (-0.567 - 2.456) | | 0.221 | |
| Total tissue fat, % | 0.678 | (-0.837 - 2.192) | 0.380 | 0.986 | (-0.570 - 2.542) | | 0.214 | |
| Fat body Weight, % | 0.006 | (-0.009 - 0.021) | 0.401 | 0.009 | (-0.006 - 0.025) | | 0.238 | |
| Limb fat body Weight, % | -0.002 | (-0.008 - 0.004) | 0.515 | -0.001 | (-0.007 - 0.005) | | 0.654 | |
| Trunk fat body Weight, % | 0.009 | (-0.001 - 0.019) | 0.068 | 0.011 | (0.001 - 0.021) | | 0.038 | |
| Lean body Weight, % | -0.001 | (-0.014 - 0.012) | 0.865 | -0.008 | (-0.021 - 0.005) | | 0.230 | |
| Limb lean body Weight, % | -0.004 | (-0.009 - 0.002) | 0.163 | -0.004 | (-0.009 - 0.002) | | 0.205 | |
| Trunk lean body Weight, % | 0.004 | (-0.003 - 0.011) | 0.279 | -0.002 | (-0.009 - 0.005) | | 0.617 | |
| Limb in fat, % | -0.017 | (-0.025 - -0.008) | 0.000 | -0.017 | (-0.025 - -0.008) | | 0.000 | |
| Trunk in fat, % | 0.020 | (0.010 - 0.030) | 0.000 | 0.018 | (0.008 - 0.029) | | 0.001 | |
| Limb in lean, % | -0.005 | (-0.009 - -0.001) | 0.026 | 0.000 | (-0.005 - 0.004) | | 0.951 | |
| Trunk in lean, % | 0.007 | (0.003 - 0.011) | 0.001 | 0.003 | (-0.001 - 0.007) | | 0.133 | |
| Tested by Mann-Whitney U test or Kruskal-Wallis H test and expressed as mean (SD); DM, diabetes mellitus.  *Tested by generalized linear model and adjusted age, sex, systolic blood pressure, diastolic blood pressure, triglycerides, and HDL. (n=1277) | | | | | | | | |
