# Supplementary material for: Body composition patterns among normal glycemic, pre-diabetic, diabetic health Chinese adults in community: NAHSIT 2013–2016
Source: PLoS One. 2020 Nov 4;15(11):e0241121. doi: 10.1371/journal.pone.0241121 (PMC7641370; doi:10.1371/journal.pone.0241121)
Supplement: S4 Table — (DOCX) [file pone.0241121.s004.docx]

**S4 Table. Body composition markers that related obesity to triglyceride glucose-waist circumference (TyG-WC) index.**

| **Markers, units** | **TyG-WC index** | | | | **P value^a^** |
| --- | --- | --- | --- | --- | --- |
|  | **≥ 850.0 (n = 236)** | | **< 850.0 (n = 1122)** | |  |
|  | **Mean** | **SD** | **mean** | **SD** |  |
| **Weight, kg** | 79.4 | 12.5 | 61.6 | 10.7 | <0.001* |
| **BMI, kg/m^2^** | 28.9 | 3.7 | 23.5 | 3.3 | <0.001* |
| **Waist, cm** | 100.2 | 7.5 | 81.1 | 8.7 | <0.001* |
| **Total fat mass, g** | 27554.9 | 7160.8 | 18258.1 | 6129.0 | <0.001* |
| **Total lean mass, g** | 48429.8 | 8595.0 | 39902.5 | 8113.1 | <0.001* |
| **Total region fat, %** | 34.9 | 6.5 | 30.1 | 8.4 | <0.001* |
| **Total tissue fat, %** | 36.1 | 6.6 | 31.3 | 8.7 | <0.001* |
| **Fat body weight, %** | 34.6 | 6.5 | 29.6 | 8.4 | <0.001* |
| **Limb fat body weight, %** | 12.5 | 3.7 | 12.5 | 4.2 | 0.826 |
| **Trunk fat body weight, %** | 21.0 | 3.5 | 16.0 | 4.7 | <0.001* |
| **Lean body weight, %** | 61.1 | 6.4 | 64.9 | 8.2 | <0.001* |
| **Limb lean body weight, %** | 27.4 | 3.5 | 28.5 | 4.1 | 0.005 * |
| **Trunk lean body weight, %** | 29.2 | 3.3 | 31.1 | 4.2 | <0.001* |
| **Limb in fat, %** | 35.7 | 5.0 | 41.8 | 6.1 | <0.001* |
| **Trunk in fat, %** | 61.0 | 4.8 | 54.0 | 6.4 | <0.001* |
| **Limb in lean, %** | 44.8 | 2.8 | 43.8 | 2.5 | <0.001* |
| **Trunk in lean, %** | 47.9 | 2.5 | 47.9 | 2.1 | 0.360 |

BMI, body mass index.

*Statistical significance as P < 0.05.

**^a^** Mann–Whitney U test
