# Supplementary material for: Body composition patterns among normal glycemic, pre-diabetic, diabetic health Chinese adults in community: NAHSIT 2013–2016
Source: PLoS One. 2020 Nov 4;15(11):e0241121. doi: 10.1371/journal.pone.0241121 (PMC7641370; doi:10.1371/journal.pone.0241121)
Supplement: S5 Table — (DOCX) [file pone.0241121.s005.docx]

**S5 Table. Body composition markers that related obesity to triglyceride glucose-waist circumference (TyG-WC) index.**

| **Markers, units** | **Pearson correlation (TyG-WC index)** | **P value** | **Regression coefficient (TyG-WC index) ^a^** | | **P value** |
| --- | --- | --- | --- | --- | --- |
|  |  |  | Beta | 95% CI |  |
| **Weight, kg** | 0.722 | <0.001* | 0.08896 | (0.08486 to 0.09306) | <0.001* |
| **BMI, kg/m^2^** | 0.752 | <0.001* | 0.03026 | (0.02883 to 0.03168) | <0.001* |
| **Waist, cm** | 0.931 | <0.001* | 0.10087 | (0.09929 to 0.10244) | <0.001* |
| **Total fat mass, g** | 0.678 | <0.001* | 56.89481 | (54.92524 to 58.86439) | <0.001* |
| **Total lean mass, g** | 0.507 | <0.001* | 28.87048 | (26.20707 to 31.53389) | <0.001* |
| **Total region fat, %** | 0.350 | <0.001* | 0.05757 | (0.05424 to 0.0609) | <0.001* |
| **Total tissue fat, %** | 0.344 | <0.001* | 0.05897 | (0.05552 to 0.06242) | <0.001* |
| **Fat body weight, %** | 0.360 | <0.001* | 0.00059 | (0.00055 to 0.00062) | <0.001* |
| **Limb fat body Weight, %** | 0.019 | 0.486 | 0.00018 | (0.00017 to 0.0002) | <0.001* |
| **Trunk fat body weight, %** | 0.600 | <0.001* | 0.0004 | (0.00038 to 0.00042) | <0.001* |
| **Lean body weight, %** | -0.289 | <0.001* | -0.00043 | (-0.00046 to -0.00039) | <0.001* |
| **Limb lean body weight, %** | -0.149 | <0.001* | -0.00015 | (-0.00016 to -0.00013) | <0.001* |
| **Trunk lean body weight, %** | -0.298 | <0.001* | -0.00022 | (-0.00024 to -0.0002) | <0.001* |
| **Limb in fat, %** | -0.613 | <0.001* | -0.00014 | (-0.00017 to -0.00012) | <0.001* |
| **Trunk in fat, %** | 0.669 | <0.001* | 0.00023 | (0.0002 to 0.00026) | <0.001* |
| **Limb in lean, %** | 0.250 | <0.001* | 0.00007 | (0.00006 to 0.00008) | <0.001* |
| **Trunk in lean, %** | -0.068 | 0.012* | -0.00003 | (-0.00004 to -0.00002) | <0.001* |

**^a^**Tested by GLM and adjusted age, sex, systolic blood pressure, diastolic blood pressure, triglycerides, HDL, and individual socioeconomic status levels. (n = 1277).
